# Expression and Function of Organic Cation Transporter 2 in Pancreas

**DOI:** 10.3389/fcell.2021.688885

**Published:** 2021-05-28

**Authors:** Sandra Schorn, Ann-Kristin Dicke, Ute Neugebauer, Rita Schröter, Maren Friedrich, Stefan Reuter, Giuliano Ciarimboli

**Affiliations:** Experimental Nephrology, Medicine Clinic D, University Hospital Münster, Münster, Germany

**Keywords:** organic cation transporters, glucose transporter, glucose, pancreas, metformin

## Abstract

Organic cation transporters (OCT) play an important role in mediating cellular uptake of several pharmaceuticals, such as the antidiabetic drug metformin and the platinum-derived chemotherapeutics. Since these drugs can also affect the pancreas, here it was investigated whether these transporters are expressed in this organ. An interaction between OCT2 and the glucose transporter 2 (GLUT2), which is expressed with important functional consequences in the kidneys and in the pancreas, has already been demonstrated elsewhere. Therefore, here it was further investigated whether the two proteins have a functional relationship. It was demonstrated that OCT2 is expressed in pancreas, probably in β cells of Langerhans islets, together with GLUT2. However, a co-localization was only evident in a cell-line model of rat pancreatic β cells under incubation with high glucose concentration. High glucose stimulated OCT2 expression and activity. On the other side, studies conducted in human embryonic kidney cells stably expressing OCT2, showed that overexpression of GLUT2 decreased OCT2 activity. Unfortunately, pull-down experiments aimed to confirm a physical OCT2/GLUT2 interaction were not successful. Renal glucose excretion was reduced in mice with genetic deletion of OCT2. Nonetheless, in these mice no regulation of known kidney glucose transporters was measured. Therefore, it may be speculated that OCT2 may influence cellular trafficking of GLUT2, without changing its amount. OCT2 may play a role in drug uptake of the pancreas, and its activity may be regulated by glucose and GLUT2. Vice versa, GLUT2 activity may be regulated through an interaction with OCT2.

## Introduction

Metformin is a broadly used antidiabetic drug with glucose-lowering effects. It seems that metformin main mechanisms of action derive from inhibition of hepatic gluconeogenesis and improved glucose uptake in skeletal muscles (Pernicova and Korbonits, 2014). Metformin has also an effect on pancreas function restoring insulin secretion and protecting pancreatic β cells from lipotoxicity or glucotoxicity (Yang et al., 2017). Cellular metformin passage through the plasma membrane is mainly mediated by organic cation transporters (OCT) and multidrug and toxin extrusion proteins (MATEs) (Zhou et al., 2016). Polymorphisms of the genes encoding for these transporters may change cellular transport of metformin, despite that the question whether these mutations have a clinical significance is still debated (Zazuli et al., 2020). Interestingly, a mutation in the *Solute Carrier 2A2* (*SLC2A2*) gene encoding for the glucose transporter 2 (GLUT2) has been demonstrated to have a clear clinical impact on metformin efficacy especially in obese patients (Zhou et al., 2016). GLUT2 is expressed in the central nervous system, in the liver, intestine, kidneys, and pancreatic islet β cells, where, at least in rodents, it is required for glucose-stimulated insulin secretion (Thorens, 2015). In enterocytes, hepatocytes, and in epithelial cells from the kidney proximal convoluted tubule epithelial cells, GLUT2 is expressed mainly in the basolateral membrane domain. However, in enterocytes after a sugar meal (Leturque et al., 2009) and in renal proximal tubules in dependence from increasing glucose concentrations (Marks et al., 2003), GLUT2 can translocate to the apical membrane. Using a mating-based split-ubiquitin-system (mbSUS), a protein-protein interaction between GLUT2 and the human OCT2 (hOCT2) was observed (Snieder et al., 2019). Because hOCT2 is a transporter for metformin (Song et al., 2008; Konig et al., 2011) and metformin also acts on pancreas (Yang et al., 2017), in this brief research report we investigated, whether OCT2 is expressed in pancreatic tissue and whether GLUT2 and glucose can regulate its activity.

## Materials and Methods

### Cells

As a model for pancreatic β cells the INS-1 832-13 cells (Duke Molecular Physiology Institute, Duke University, Durham, NC, United States) (Hohmeier et al., 2000), which are cells isolated from X-ray induced rat-insulinoma and can secrete insulin, were used. The cells were cultured in Roswell Park Memorial Institute medium [RPMI 1640, Sigma-Aldrich, Taufkirchen, Germany (Hohmeier et al., 2000)] supplemented with 10% fetal calf serum (FCS), 1% penicillin/streptomycin, 10 mM HEPES, 1 mM glutamine, 2 mM Na^+^-pyruvate and 0.05 mM 2-mercaptoethanol. In some experiments, glucose concentration was increased from 9.8 to 14.8 mM. Osmolality in control cells was adjusted by mannose addition.

The effect of transfection with human GLUT2 (hGLUT2, a gift from RESOLUTE Consortium & Giulio Superti-Furga, Addgene plasmid #132126^1^) cloned into a pcDNA DEST47 vector) was studied in human embryonic cells (HEK) stably over-expressing the human OCT2 [hOCT2, for a detailed description of these cells see Biermann et al. (2006)]. Transfection was performed using the calcium phosphate transfection method. 72 h after transfection with GLUT2 or the empty vector (EV), the cells were used for ASP^+^ uptake measurements and western blot analysis, as described below.

Immunoprecipitation experiments with lysates from HEK cells transfected with hOCT2 tagged with green fluorescent protein (GFP) at the carboxy-terminus (hOCT2-GFP) (as described in Brast et al., 2012) and GLUT2 were performed using GFP trap agarose beads (ChromoTek, Planegg-Martinsried, Germany). Briefly, cells were lysed with Laemmli buffer [10% SDS, 20% (v/v) Glycerol, 125 mM Tris–HCl (pH 6.8)] containing 200 mM dithiothreitol and 0.004% bromophenol blue. Lysates were incubated 2.5 h at 4°C under rotation with GFP trap agarose beads. The immunocomplexes bound to the beads were recovered by centrifugation, washed five times, resuspended in Laemmli buffer, denaturated at 95°C for 5 min, and loaded onto 4–20% SDS-polyacrylamide gels (Mini-Protean TGX gels, Bio-Rad, Hercules, CA, United States). For western blot analysis the proteins on the gel were transferred to a nitrocellulose membrane incubated with blocking reagent [3% milk powder and 1% bovine serum albumin (BSA) in Tris-buffered saline with Tween20 (TBST)]. After incubation with the primary antibody (1:500 rabbit-polyclonal antibody to hOCT2, Sigma-Aldrich, Munich, Germany, or 1:1000 rabbit-polyclonal antibody to hGLUT2 Proteintech, St. Leon-Rot, Germany, or 1:1000 rabbit antibody against α-actinin, Cell Signaling Technology, Frankfurt, Germany), membranes were incubated with anti-rabbit HRP-conjugate (1:10,000 Thermo Fisher Scientific, Waltham, MA, United States), then covered with Lumi-Light or Lumi-Light Plus (Roche, Mannheim, Germany) before exposure (c600 azure biosystem imager, Azure Biosystems, Dublin, CA, United States).

### Fluorescence Measurements

The fluorescent organic cation ASP^+^ was used as a tracer of OCT-mediated transport, as already described in detail (Wilde et al., 2009; Wittwer et al., 2013). Measurements were performed using a microplate fluorescence reader with excitation at 465 nm and emission at 590 nm (Infinite F200, Tecan, Switzerland) (Wilde et al., 2009). ASP^+^-concentration was 1 μM in experiments with hOCT2-expressing HEK cells and 5 μM for experiments with INS-1 cells.

### PCR Analysis

For PCR analysis, total RNAs were isolated using the Qiagen RNeasy Minikit (Qiagen, Gilden, Germany) and reverse transcription was performed using the Superscript II system (Invitrogen, Carlsbad, CA, United States), both according to the manufacturer’s recommendations. Standard PCR was performed using specific primer pairs as listed in Supplementary Table 1. The PCR products were separated using agarose gel electrophoresis. The bands were sequenced for confirmation of amplification product identity.

### Animals

Male OCT2^–/–^ mice (Prof. Schinkel, The Netherlands Cancer Institute, Amsterdam, Netherlands) on Bl6 background, and male Bl6 wild-type (WT) mice approximately 9 weeks old and weighing 25–30 g were used. Compared with WT animals, OCT2^–/–^ mice display no obvious phenotypic abnormalities (Jonker et al., 2003). Experiments were approved by a governmental-committee on animal welfare (84-02.04.2014.A454) and were performed in accordance with national animal protection guidelines. Mice were placed in metabolic cages and 24 h urines were collected for the determinations of glucose excretion. Glucose was measured by the hexokinase method with a Roche Hitachi Modular automatic analyzer (Gluco-quant Glucose, Roche, Mannheim, Germany). In some experiments, pancreas tissues from mice and rats were collected. For immunofluorescence analysis, pancreas was removed and fixed with 4% neutral buffered formalin. Expression of OCT2 and GLUT2 in mouse pancreas was investigated using specific antibodies raised against mouse OCT2 (1:300, a generous gift from Prof. Koepsell) and against GLUT2 (1:50, Glut2 C19 SC-7580, Santa Cruz Biotechnology, Dallas, TX, United States). Five micrometer thick cryosections were prepared from mouse pancreas, which have been fixed in melting 2-methylbutane. Cryosections were incubated with 0.2% Triton in phosphate-buffered saline (PBS) for 5 min at room temperature, three times washed with PBS and then incubated 1 h with a 1:1 mix of 10% BSA and 10% normal human serum (NHS) in PBS. After this, sections were incubated overnight at 4°C with primary antibodies. After washing in PBS, the sections were incubated for 45 min at room temperature in the dark with PBS-diluted secondary antibodies (donkey Alexa Fluor 488 anti- goat -Ig, and Alexa Fluor 647 anti- rabbit -Ig Invitrogen, 1:1,000). Sections were rinsed with PBS, coverslipped with Fluoromount aqueous mounting medium (Sigma-Aldrich, München, Germany) and evaluated by epifluorescence microscopy (Observer Z1 with Apotome, Zeiss). Negative control slides were included without addition of primary antibody.

## Reagents

4-(4-dimethylaminostyril)-N-methylpyridinium (ASP^+^) was purchased from Fischer Scientific. All other reagents were of the highest purity and obtained from Sigma-Aldrich (Sigma-Aldrich, Merck Chemicals, Darmstadt, Germany).

### Statistical Analysis

Experimental data are presented as means ± SEM, with n referring to the number of totally measured replicates obtained in at least three independent experiments. Significant differences were calculated using unpaired Student’s *t*-test or ANOVA with Tukey’s post-test for multiple comparisons. A *p*-value < 0.05 was considered statistically significant. Analyses were performed using GraphPad Prism, Version 5.3 (GraphPad Software, San Diego, CA, United States).

## Results

**FIGURE 1:**
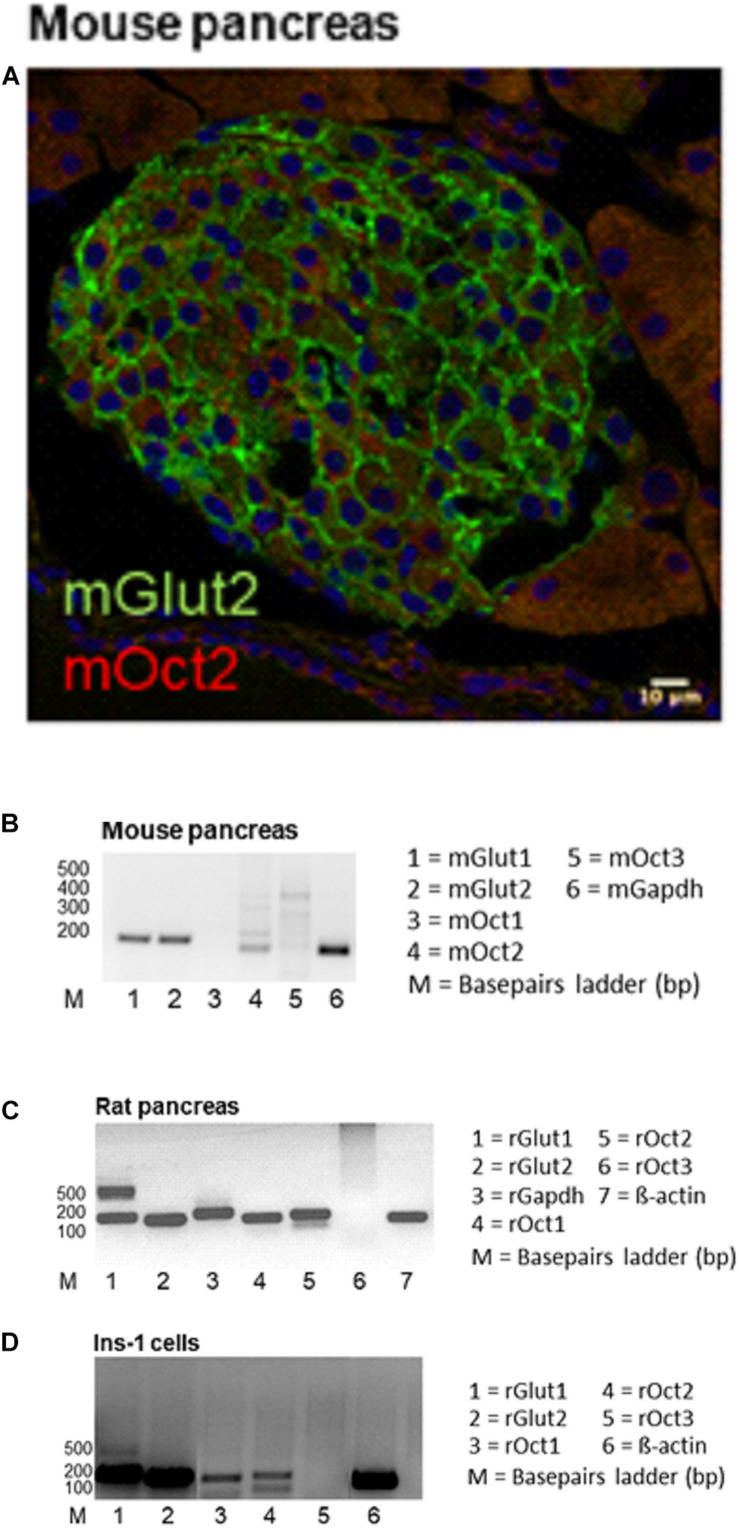
This figure shows the expression of GLUT and OCT in the pancreas. In panel **(A)** a representative immunofluorescence analysis of GLUT2 (green) and OCT2 (red) expression in a mouse pancreatic islet is shown. GLUT2 is evident in the plasma membrane of islet cells. OCT2 seems to be expressed in intracellular compartments of some islet cells. No GLUT2/OCT2 co-localization is observed. Panel **(B)** shows that mouse pancreas expresses mRNA of GLUT1 and 2 and of OCT2, but not of OCT1 and 3. For OCT2 several bands are visible, but sequencing confirmed the identity of the lower band with OCT2. In rat pancreas **(C)** OCT expression became visible only after reamplification. However, this reamplification caused the appearance of an unspecific band for GLUT1. Sequencing confirmed the lower band to be specific for GLUT1. The INS-1 cells show the same transporter expression pattern as the rat pancreas **(D)**.

**FIGURE 2:**
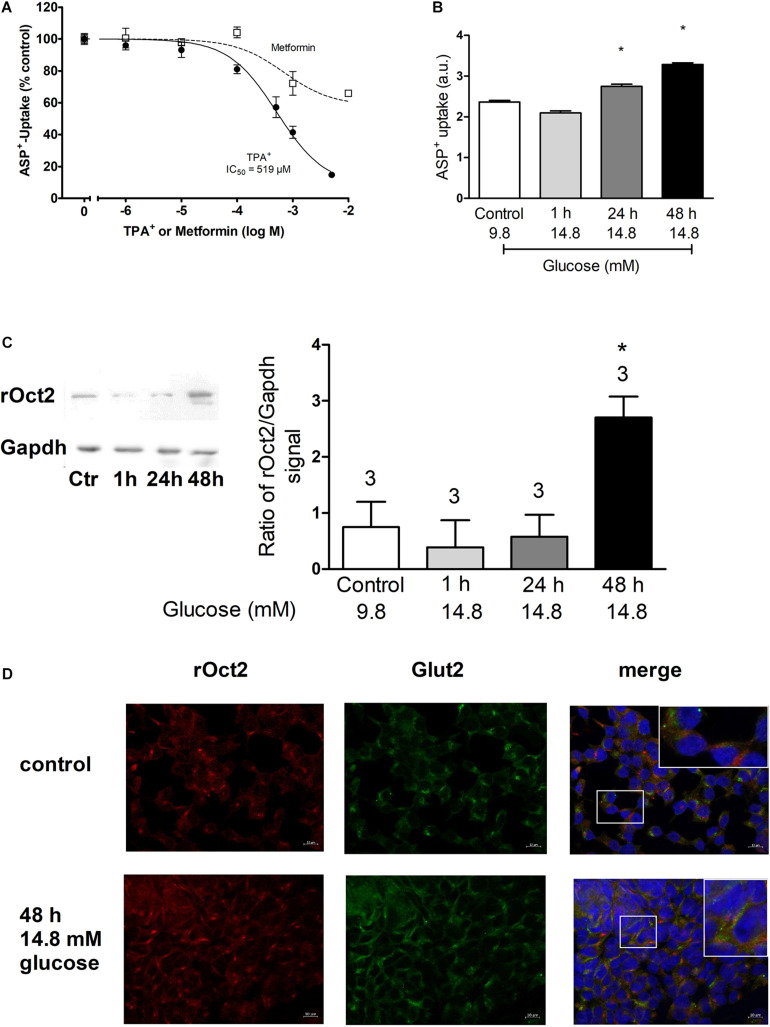
This figure shows the ASP^+^ transport characteristics of INS-1 cells and the analysis of OCT2 and GLUT2 expression in dependence from glucose concentration. Panel **(A)** shows that TPA^+^ inhibits concentration-dependently the ASP^+^ uptake with an IC_50_ of 519 μM. Inhibition of ASP^+^ uptake by metformin is also shown (at least six replicates for concentration measured in three independent experiments). Panel **(B)** shows the effect of incubation with 14.8 mM glucose for 1, 24, or 48 h on the ASP^+^ uptake in arbitrary units (a.u.) of INS-1 cells. Control experiments were performed with a glucose concentration of 9.8 mM. Osmolarity was adjusted in control experiments by adding mannose. High glucose significantly stimulated ASP^+^ uptake after incubation for 24 and 48 h (ANOVA with Tukey’s post-test, nine replicates measured in four independent experiments). Panel **(C)** shows the evaluation of western blot analysis of OCT2 expression under incubation with high glucose. Only after 48 h incubation with 14.8 mM glucose a significant increase of OCT2 expression was observed (*, ANOVA with Tukey’s post-test, three independent experiments). The insert on the left shows an example of the western blot analysis. Panel **(D)** shows the immunofluorescence analysis of OCT2 (red) and GLUT2 (green) expression in INS-1 cells after 48 h incubation with normal (9.8 mM) or high (14.8 mM) glucose. The labeling of nuclei with 4′,6-diamidino-2-phenylindole (DAPI, blue) is also shown. In the upper right corners of the overlay picture a magnification of the field indicated by a box is shown.

Expression of GLUT2 and OCT2 was studied in pancreas tissues from mice, rats and in the INS-1 cells (Figure 1). Immunofluorescence analysis of protein expression in mouse pancreas shows that islets express GLUT2 in the plasma membrane, while OCT2 is visible only in some cells in intracellular compartments (Figure 1A). No co-localization of the two proteins was evident. Mouse pancreas expresses mRNA for GLUT1, GLUT2, and OCT2 (Figure 1B). For OCT2 more bands were detected. The thicker lower one corresponds to mOCT2, as confirmed by sequencing (the identity of all PCR-products was confirmed by sequencing). No specific band for OCT1 and OCT3 was detected. In rat pancreas (Figure 1C) an expression of OCT1- and OCT2-, but not of OCT3-mRNA was evident only upon reamplification of the PCR-products, which produced a double band for GLUT1. This suggests that OCT1- and 2-mRNA expression is relatively low. For GLUT1, only the lower band is specific. GLUT2-mRNA is also robustly expressed in rat pancreas. Since the INS1-cells show a similar transporter expression pattern (Figure 1D, also here a reamplification was necessary to detect OCT1- and OCT2-mRNA, the thicker band was specific for the transporters as revealed by sequencing), it was investigated, whether these cells show any ASP^+^-transport. INS-1 cells are able to take up ASP^+^ (Figure 2A): this uptake was inhibited concentration-dependently by tetrapentylammonium (TPA^+^), a known OCT-inhibitor (Zhang et al., 1999; Wilde et al., 2009), with an apparent affinity (IC_50_) of 519 μM. Metformin, which is well known to interact with OCT, showed a lower but still significant inhibitory capacity than TPA^+^. Increasing glucose concentration from 9.8 to 14.8 mM resulted in an increased uptake of ASP^+^ after 24 and 48 h incubation (Figure 2B). Osmolality was equilibrated by mannose addition. Western blot analysis of the cells showed that rat OCT2 protein expression significantly increased after 48 h incubation with high glucose (Figure 2C). Immunofluorescence analysis of INS-1 cells after 48 h incubation with 14.8 mM glucose suggests an increased cell proliferation (as already shown in Olson et al., 1998), and an increased expression of OCT2 and GLUT2, with augmented distribution to the plasma membrane (Figure 2D). In some cells, a partial co-localization of OCT2 and GLUT2 can be observed after 48 h incubation with 14.8 mM glucose (Figure 2D, merge).

**FIGURE 3:**
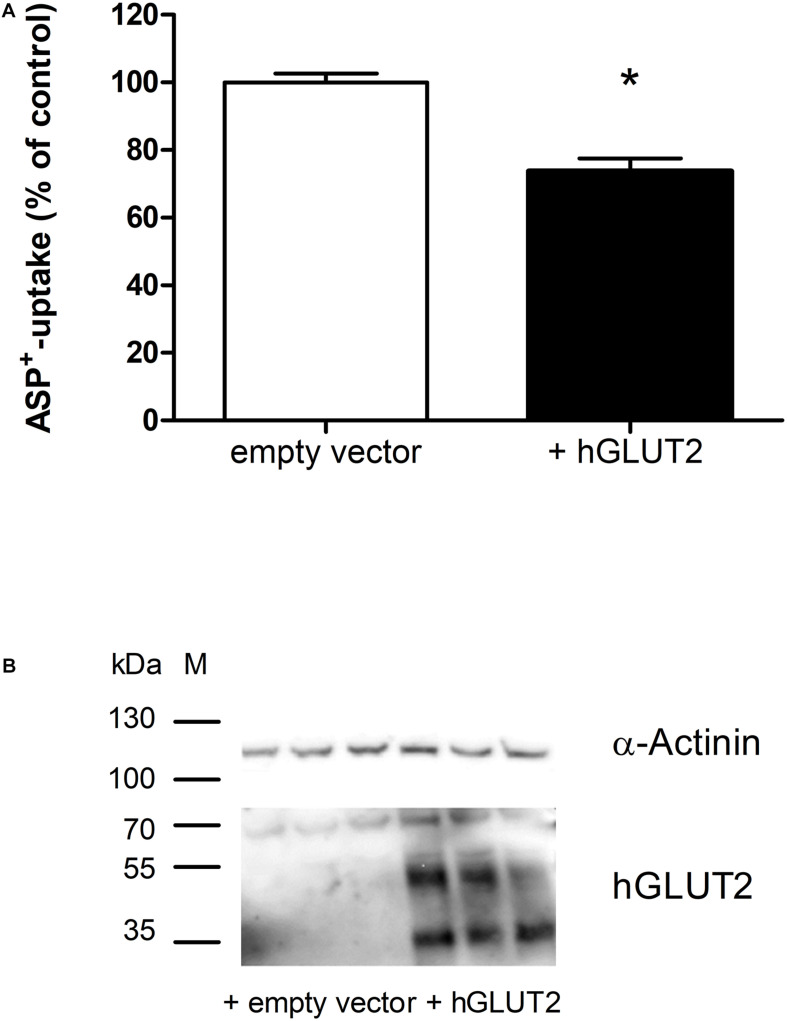
Transfection of HEK cells stably expressing hOCT2 with GLUT2: effects on the ASP^+^ uptake. Panel **(A)** shows the uptake of 1 μM ASP^+^ in HEK cells 72 h after transfection with GLUT2 or empty vector (EV). Overexpression of GLUT2 significantly inhibited ASP^+^ uptake (*, unpaired *t*-test, 27 replicates measured in three independent experiments). Panel **(B)** shows the western blot analysis of GLUT2 72 h after transfection. α-Actinin was used as loading marker. This antibody against hGLUT2 is known to detect mature glycosylated hGLUT2 at 60-70 kDa, non-glycosylated hGLUT2 at 38–45 kDa, and a hGLUT2 fragment at around 35 kDa (https://www.ptglab.com/products/SLC2A2-Antibody-20436-1-AP.htm). All these bands are visible in this western blot analysis, especially in HEK cells transfected with hGLUT2, confirming that transfection with hGLUT2 was successful. Bands corresponding to GLUT2 were observed mainly in transfected cells.

Transfection of hGLUT2 in HEK cells overexpressing hOCT2 caused a significant inhibition (−26 ± 4%) of hOCT2 function measured as ASP^+^-uptake (Figure 3A). The transfection with hGLUT2 resulted in an evident GLUT2 protein expression (Figure 3B). The expression levels of hOCT2 were not changed (not shown). However, GLUT2 was not detectable in GFP-trap pull-down experiments (not shown).

**FIGURE 4:**
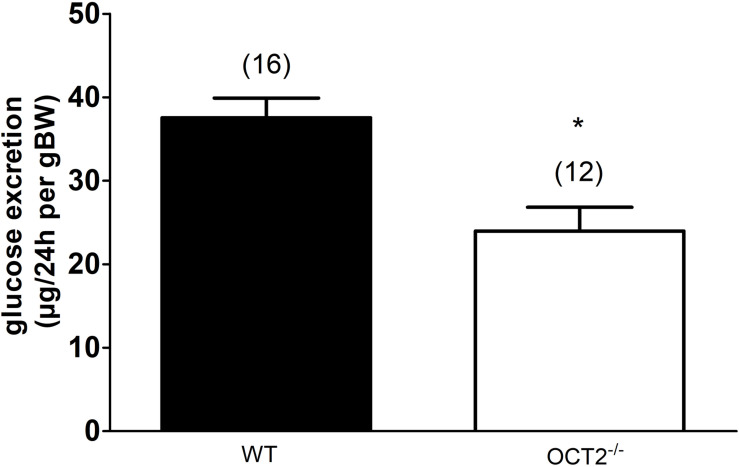
Glucose excretion measured in urines from WT- and OCT2^–/–^-mice. The mice were placed in a metabolic cage and urine was collected over 24 h. The glucose concentration is normalized according to the weight of the animal (body weight, BW). OCT2^–/–^-mice excrete significantly less glucose than the WT animals (*, unpaired *t*-test). The numbers on the top of the columns indicate the number of mice used in the experiments.

The *in vitro* experiments suggest that high glucose and also GLUT2 protein expression can change OCT activity and cellular distribution. Since high amounts of GLUT2 and OCT2 are expressed in the basolateral plasma membrane domain of renal proximal tubules (Koepsell et al., 2007; Thorens, 2015), we tried to investigate whether OCT2 influences glucose kidney handling by comparing renal glucose excretion in WT- and OCT2^–/–^-mice (Figure 4). A decreased glucose urinary secretion was measured in OCT2^–/–^-compared to WT mice (Figure 4).

## Discussion

In this brief research report, it has been demonstrated that OCT1 and 2 are expressed in the pancreas. Previously, expression of OCT1 was demonstrate in human pancreatic stellate cells (Wu et al., 2018). Focusing on OCT2, it seems to be expressed in some cells of the Langerhans islets. From experiments with the INS-1 cells, which are able to release insulin and are a model for β cells, it can be speculated that OCT2 is expressed in the β-cells. Interestingly, a partial co-localization with GLUT2 was only detected under incubation with high glucose concentration, suggesting that probably in this cell system, a protein conformational change may be induced by high-glucose, which then facilitates OCT2-GLUT2 interaction. Increased OCT2 expression and function under incubation with high-glucose may facilitate metformin action under diabetic conditions. INS-1 cells can transport ASP^+^ and this transport can be inhibited by TPA^+^ with an apparent affinity, which is much lower than that observed using rat OCT1 (rOCT1) (Ciarimboli et al., 2005) or rOCT2 (Wilde et al., 2009) in expression systems. This suggests that the apparent affinity can be changed by specific cellular regulation pathways (Ciarimboli et al., 2004). In INS-1 cells, a low but significant inhibition of ASP^+^ uptake by metformin was measured. This inhibition is lower than that measured using ASP^+^ with cloned OCT (see Frenzel et al., 2019), again suggesting an influence of cell specific factors in determining transport characteristics.

Transfection of GLUT2 decreases the transport activity of hOCT2 overexpressed in HEK cells, suggesting that the hOCT2-GLUT2 interaction detected using the mbSUS (Snieder et al., 2019) may have functional consequences. However, in pull-down experiments using GFP-trap and HEK cells transfected with hOCT2-GFP [the GFP tag is localized at the carboxy-terminus of the transporter and does not change its function, as already demonstrated in Brast et al. (2012)] it was not possible to confirm a direct hOCT2-GLUT2 interaction. It can be speculated, that the interaction domain is close to the carboxy-terminus, where hOT2 bears the GFP-tag. The tag may change the conformation of this part of the protein, hindering the interaction with GLUT2.

Interestingly, genetic deletion of OCT2 in mice caused a significant lower excretion of glucose compared to WT animals. In a previous work, performing a proteomic analysis of kidneys from WT- and OCT1/2^–/–^ mice no different expression of proteins involved in glucose resorption [sodium glucose transporter (SGLT)1 and 2, GLUT1 and 2] was detected (Hucke et al., 2019), suggesting that the difference in glucose excretion is not due to changes in global protein amount. Perhaps the OCT2/GLUT2 interaction changes the transport characteristics of these proteins or modify their trafficking from/to the plasma membrane. For example, in the absence of OCT2, GLUT2 may increase its transport activity or/and may traffic to the apical plasma membrane, in both ways increasing renal glucose reabsorption. Unfortunately, we were not able to analyze these aspects in the kidneys.

Platinum derivatives such as the chemotherapeutic drugs cisplatin and oxaliplatin, are also substrates of OCT (Sprowl et al., 2013). Anticancer treatment with these drugs is well known to cause severe side-effects such as nephrotoxicity and peripheral neurotoxicity, which are probably mediated by OCT-mediated uptake (Hucke et al., 2019; Sprowl et al., 2013). There is also some evidence that cisplatin and oxaliplatin can cause pancreatic toxicity (Komdeur et al., 2007; Yadav, 2019). Therefore, it may be speculated that OCT2 may play a role also in this type of unwanted side-effect of platinum-based chemotherapy.

Organic cation transporters have a large binding pocket, with specific interaction domains for different substrates. Therefore, they are polyspecific, meaning that they can accept many different substances as substrate (Koepsell et al., 2007). For this reason, OCT can mediate the cellular uptake of many different drugs and can be involved in cellular drug toxicity. While the OCT-mediated drug toxicity in organs like the kidneys and the liver is well known (Motohashi and Inui, 2016), pancreatic drug toxicity has not yet been associated with OCT expression. Many medications, which are associated with pancreas toxicity (Jones et al., 2015) are substrates of OCT, like prazosin, procainamide, ranitidine, and cimetidine (Koepsell, 2020).

## Conclusion

In conclusion, here we demonstrated that OCT2 is expressed in the pancreas and that high-glucose stimulates its expression and co-localization with GLUT2. A possible functional role of this interaction is plausible, but we were not able to define the interaction mechanisms. Further studies are necessary to elucidate the characteristics of this interaction and whether it may have some functional implication.

## Data Availability Statement

The raw data supporting the conclusions of this article will be made available by the authors, without undue reservation.

## Ethics Statement

The animal study was reviewed and approved by Landesamt für Natur, Umwelt und Verbraucherschutz Nordrhein-Westfalen.

## Author Contributions

SS, A-KD, UN, RS, MF, SR, and GC performed and evaluated the experiments. GC planned the study and wrote the manuscript. All authors contributed to the article and approved the submitted version.

## Conflict of Interest

The authors declare that the research was conducted in the absence of any commercial or financial relationships that could be construed as a potential conflict of interest.

## Footnotes

1 http://n2t.net/addgene:132126
