# Supplementary material for: Expression and Function of Organic Cation Transporter 2 in Pancreas
Source: Front Cell Dev Biol. 2021 May 28;9:688885. doi: 10.3389/fcell.2021.688885 (PMC8195675; doi:10.3389/fcell.2021.688885)
Supplement: Supplementary file 1 [file Data_Sheet_1.PDF]

**Supplementary Material Table 1. Primer sequences**

| Primers for | | Sequence (5' - 3') | Product length [bp] |
|---|---|---|---|
| mouse (m) Glut1 | Sense | CGATCTGAGCTACGGGGTCT | 177 |
| | Antisense | CCTCCCACAGCCAACATGAG | |
| mGlut2 | Sense | GTGCTGCTGGATAAATTCGCC | 180 |
| | Antisense | ATTGCAGACCCAGTTGCTGA | |
| mOct1 | Sense | GGCCGCATCTACCCAATAGC | 113 |
| | Antisense | CCAAGACAAGCGAGGGTCAC | |
| mOct2 | Sense | TTCAGAGCCTGACGGCAGAT | 151 |
| | Antisense | TGCATGATGAGGCCCTGGTA | |
| mOct3 | Sense | TGACTGGCGCTATGTGGAGA | 193 |
| | Antisense | GCCAACGCCGAAACAGGATA | |
| mβ-Actin | Sense | GCCTCACTGTCCACCTTCCA | 109 |
| | Antisense | CAGCTCAGTAACAGTCCGCCT | |
| mGapdh | Sense | TGGCCTTCCGTGTTCCTACC | 147 |
| | Antisense | GGTCCTCAGTGTAGCCCAAGATG | |
| rat (r) Glut1 | Sense | GCTGTGGCTGGCTTCTCTAA | 185 |
| | Antisense | CCGGAAGCGATCTCATCGAA | |
| rGlut2 | Sense | CGCAACATGTCAGAAGACAAGATCA | 162 |
| | Antisense | GTCATCCAGAGGAACACCCAAA | |
| rOct1 | Sense | CAAACAGGCAAGTCCTCAAGTAC | 154 |
| | Antisense | GGTTGGTAGTTCATTTGGAACC | |
| rOct2 | Sense | GCAAGCAGACCGTCCGCTAAG | 173 |
| | Antisense | CAGACCGTGCAAGCTACAGCAC | |
| rOct3 | Sense | TCTACTTCTAATGTCTGAGGCCC | 168 |
| | Antisense | GGTAGCATGATAAATCTTTAT | |
| rβ-Actin | Sense | CTGTGTGGATTGGTGGCTCT | 135 |
| | Antisense | CAGCTCAGTAACAGTCCGCC | |
| rGapdh | Sense | CATCAACGACCCCTTCATTGAC | 197 |
| | Antisense | ACTCCACGACATACTCAGCACC | |

| | | | |
|---|---|---|---|
| human (h) GLUT2 | Sense | GCCACACTCACACAAGACCTGG | 119 |
| | Antisense | AACTGGAAGGAACCCAGCACA | |
| hOCT2 | Sense | CGCCATTCCTGGTCTACCGGC | 145 |
| | Antisense | GCTTCCTCGATGGTCTCAGGC | |
| hGAPDH | Sense | CAAGCTCATTTCCTGGTATGAC | 179 |
| | Antisense | GTGTGGTGGGGGACTGAGTGTGG | |
